# A Remarkable Case of Numerous Births; with Observations

**Published:** 1789

**Authors:** Maxwell Garthshore


VI. *A remarkable Case of numerous Births; with Observations. By* Maxwell Garthshore, *M. D. F. R. S. and A. S. in a Letter to Sir* Joseph Banks, *Bart. P. R. S.* Vide *Philosophical Transactions of the Royal Society of London, Vol.* LXXVII. *for the Year* 1787. *Part* II. 4to. London, 1787.

THE account of this extraordinary case is furnished by Mr. John Hull, Surgeon at Blackburn, in Lancashire. The subject of it was a poor woman named Margaret Waddington, aged twenty-one years, of Lower Darwin, near Blackburn. She had formerly been delivered of one child at the full term of pregnancy, and conceived a second time about the beginning of December, 1785. At the end of the third month of this second pregnancy she thought herself quite as large as she had formerly been in the ninth, and she had for some time been much troubled with nausea, vomiting, and pain of her loins, to which was now added a distressing shortness of breath. Towards the middle of April, 1786, all these complaints were much increased; she had much tension and pain over the abdomen; her vomiting was incessant, and

ſhe now could not make water but with the utmoſt difficulty. On the 24th of April, at which time ſhe was ſuppoſed to have arrived at the twentieth week of pregnancy, ſhe was ſeized with labour pains. The next day Mr. Hull was ſent for, and ſhe was ſoon delivered of a ſmall, dead fœtus. The pains continuing, this was ſoon followed by a ſecond: to this very ſoon ſucceeded a third, larger than the firſt, which was alive: after theſe a fourth followed, that was very putrid; and, laſtly, a fifth, larger than any of the former, and alive. Theſe five children were all females; two of them, as we have ſeen, were born alive; and the whole operation, Mr. Hull obſerves, was performed in the ſpace of fifty minutes. Each child, we are told, preſented naturally, was preceded by a ſeparate burſt of water, and was delivered by the natural pains only. The placenta was uncommonly large, and not divided into diſtinct placentulæ, but conſiſted of one uniform cake. Each funis was contained in a ſeparate cell, within which each child had been lodged; and, Mr. Hull obſerves, it was eaſy to perceive, by the ſtate of the funis, and that part of the placenta to which it adhered, in which ſac the dead, dead, and in which the living children had been contained.

The two living children having ſurvived their birth but a ſhort time, were preſerved by Mr. Hull, together with the other three, in ſpirits *, and having weighed and meaſured them, he found their proportions to be as follows in averdupois weight, inches and parts :

| | Oz. | Dr. | | Inches. |
|---|---|---|---|---|
| The 1ſt born dead | 6 | 12 | Length | 9 |
| The 2d —— putrid | 4 | 6 | ——— | $8\frac{1}{4}$ |
| The 3d —— alive | 8 | 12 | ——— | $9\frac{1}{8}$ |
| The 4th —— putrid | 6 | 12 | ——— | $9\frac{1}{8}$ |
| The 5th —— alive | 9 | — | ——— | $9\frac{1}{8}$ |

The mother recovered without any accident. Mr. Hull concludes his account with obſerving that the huſband of this poor woman had been in an infirm ſtate of health for three years paſt, and at the time ſhe was delivered laboured under a confirmed phthiſis.

Dr. Garthſhore, in his obſervations on numerous births, annexed to Mr. Hull's paper, has brought together a great number of curious facts relative to this ſubject.

* They are now depoſited in the muſeum of Mr. John Hunter.

He

He ſtates, that, at the Britiſh Lying-in Hoſpital, where 18,300 women have been delivered, the proportion of twins has been only once in 91 births; that, in the Weſtminſter General Diſpenſary, of 1897 women delivered, the proportion of twins has been once in 80 births; but that in the Dublin Lying-in Hoſpital, where upwards of 21,000 women have been delivered, the proportion of twins has been once in 62 births. The average of theſe different numbers will be found to be about once in 78 births.

Dr. Garthſhore obſerves, that the calculations made in Germany from great numbers, in various ſituations, ſtate twins as happening in a varied proportion from once every 65th to once every 70th time; but at Paris, as he learns from an accurate calculation made by M. Tenon, in 104,591 births, the proportion of twins has been only once in 91, which is only a ſmall degree leſs than the calculation at the Britiſh Lying-in Hoſpital.

It would be eaſy, he obſerves, to add other calculations, all differing from the above and from one another; but theſe, he imagines, will be ſufficient to ſhow that nature obſerves no certain rule in this matter, and that even twins, the moſt uſual variation, is not a very common occurrence.

He remarks, that when we advance to triplets, or three born at once, we find, comparatively, very few inſtances in this or any other country: thus, he tells us, that, at the Britiſh Lying-in Hoſpital, not one ſuch caſe has occurred; and that at the Weſtminſter General Diſpenſary it has happened only once; but he finds that in the Dublin Lying-in Hoſpital, in 21,000 births, triplets have occurred thrice, or once in 7000 times.

The author himſelf, in a pretty extenſive practice of more than thirty years in midwifery, has attended but one labour where three children were born, and is perſonally acquainted but with one lady who, at Dumfries, after bearing twins twice, was delivered of three children at once. It ſeems, however, that Dr. Hamilton, Profeſſor of Midwifery at Edinburgh, has, in leſs than twenty-five years, ſeen triplets born there five or ſix times.

Mauriceau, who paſſed a long life in very extenſive practice, at Paris, met with inſtances of three children at once only a few times, and heard of four at a birth, in that city, but once. One circumſtance mentioned by this writer is quoted quoted by Dr. Garthſhore, as it accords with one ſomewhat ſimilar mentioned by Mr. Hull, viz. " That the huſband of one of thoſe wo-" men who bore three children was by trade a " painter, and had been, for two years prece-" ding this birth, paralytic over one half of " his body, and yet had no reaſon to doubt the " fidelity of his wife."

Theſe facts, our author obſerves, as far as they are to be depended on, may ſhew us, that the capacity of procreation in the male may remain under very infirm health; and that we ought to judge with candour of ſuch wives as are fruitful when living with very ailing huſbands, and who produce healthy children in the eighth or even ninth month after their death, as we can never ſay determinately under what degree of diſeaſe the male is totally incapable of procreation, more eſpecially as we are very certain that the female is not, when labouring under very deſperate and certainly fatal diſeaſes, provided the principal organs of generation be ſound. Nay, in caſes of pulmonary phthiſis, the life of the female ſeems to be protracted by pregnancy; and he tells us he has attended a lady, who, after being pronounced irrecoverably hectic, lived long enough to be twice delivered livered naturally of healthy children at the full time.

Our author next considers the occurrence of four born at once, which he finds to be so uncommon, that he thinks Haller's conjecture, rather than calculation, of its happening once in 20,000 births, very much under-rated, as it appears that once in 100,000 would be much nearer the truth. Of this, however, he observes, we have several well-authenticated cases which have happened in this island. In the year 1674 there was published, it seems, in London, a quarto pamphlet, entitled, "The fruitful "Wonder, or a strange Relation, from King-"ston upon Thames, of a Woman who, on "Thursday and Friday, the fifth and sixth "Days of this Instant March, 1673-4, was de-"livered of four Children at one Birth, viz. "three Sons and one Daughter, all born alive, "lusty Children, and perfect in every Part, "which lived twenty-four Hours, and then "died all much about the same Time; with "several other Examples of numerous Births, "from credible Historians, with the physical "and astrological Reasons for the same. By "J. P. Student in Physic."

Dr. Plott, in his Hiſtory of Staffordſhire, p. 194, mentions Eleanor, the wife of Henry Diven, of Watlington, who was delivered of four children at a birth in the year 1675.

Sir Robert Sibbald, in his *Scotia Illuſtrata*, after mentioning a caſe of three born at once, adds, "Imo in variis regni locis repertæ ſunt "mulieres quæ quatuor fœtus uno partu edide-"runt;" but makes no mention of more.

From the Gentleman's Magazine Dr. Garthſhore has collected the following inſtances of four children at a birth:

"Ann Boynton, of Henſbridge, in Somer-"ſetſhire, was this day, June 1, 1736, deli-"vered of three daughters and one ſon; one "of the daughters died, the reſt are likely to "live. The mother has been married but four "years, and has had twice twins before, which "completes the number of eight children at "three births."

"October 3, 1743, at Rate, in Berkſhire, "Joan Galloway was delivered of two boys "and two girls, three of whom were alive."

"In January, 1746, the wife of Plumer, a "labouring man, at Mill-Wimley, near Hit-"chin, Hertfordſhire, was delivered of three "living boys and one dead."

"Auguſt

"August 22, 1746, the wife of Williams,
" of Coventry Street, Piccadilly, was delivered
" of two boys and two girls, all likely to
" live."

"June, 1752, a woman in the parish of
" Tillicultrie, near Stirling, in Scotland, was
" delivered of four children, which were all
" immediately baptised, and all died at the
" same time next morning."

"In September, 1757, a poor woman, of
" Burton Ferry, Glamorganshire, was deliver-
" ed of three boys and a girl."

Dr. Hamilton, before mentioned, relates, that, not many years ago, a woman was delivered of four children, at Pennycuick, the seat of Sir John Clark, Bart. near Edinburgh, when she was advanced to the middle of her last month of pregnancy, and that some of these children lived two or three years. He farther says, that, five years ago, he attended a woman at Edinburgh, who, in the seventh month of her pregnancy, after a journey of thirty miles, was suddenly delivered of four children, all perfect and well grown for the time, of which one was born dead, and three alive; but those three died next day. He farther adds, that these are the only cases of quadruplets, or any larger number, he had ever heard of, as born in Scotland, in his memory.

Though cafes fimilar to the prefent, of five children born at once, are ftill much more uncommon; and though Haller's affertion of their not happening above once in a million of births, may, as our author thinks, be reckoned a very moderate calculation, yet, he obferves, we are not altogether without fome fuch inftances in this country.

In the Gentleman's Magazine he has found, that on the 5th of October, 1736, a woman, at a milk cellar in the Strand, was delivered of three boys and two girls at a birth; and that in March, 1739, at Wells, in Somerfetfhire, a woman was delivered of four fons and a daughter, all alive, all chriftened, and all then feeming likely to live.

In the *Commercium Literarium Norimbergenfe*, for the year 1731, he has met with two fimilar cafes; and he has been told by two foreign profeffors who were lately in London, that they had each of them heard of an inftance of five children born at once. One of thefe inftances is faid to have happened near Paris, and the other in the neighbourhood of Ghent.

Dr. Garthfhore very candidly obferves, that when when we advance farther in our inquiries on this ſubject we get into the region of tradition and improbability; and he has not thought it right to trouble a Society, whoſe profeſſed objects are truth and ſcience, with the numerous and wonderful relations which many grave and learned authors have recorded as facts they themſelves believed. He has, therefore, contented himſelf with juſt mentioning the caſe of a lady, who is ſaid, by Ambroſe Paré, to have brought forth ſix children at a birth; and another related by Borellus, in the ſecond century of his obſervations, of a lady, who, in the year 1650, produced at one birth eight perfect children.

Our author has thought it unneceſſary to purſue this inquiry farther; but he obſerves that the preſent is the only caſe he has found where the children were all females; that the males have in all the other caſes been at leaſt equal, and generally the moſt numerous; that in many of them, at leaſt a part was dead born; and that moſt commonly the reſt died in a ſhort time. It is thence, he thinks, clear, that theſe numerous births are certainly unfavourable to population, as very few indeed of thoſe children can be carried to near the full term of pregnancy, and fewer ſtill to that degree of ſtrength that admits admits of their being reared, where more than two are born at one time.

As a farther probable proof of the fruitlefs profufion and wafte of the human race thefe numerous births muft occafion, Dr. Garthfhore refers to the very curious experiments and obfervations lately publifhed by Mr. Hunter relative to the extirpation of the ovaria, which tend to fhow that a certain determined number of ova, capable of male impregnation, are originally formed in each ovarium; which number, when exhaufted, the female conftitution has no power to renew.

From the united teftimony of all the facts he has collected on this fubject, our author thinks it is clear, that the females of the human fpecies, though moft commonly uniparous, are, in certain circumftances to us unknown, every now and then capable of very far exceeding their ufual number; and, he adds, that it does not appear that we can fet any bounds to the powers of nature in that refpect, or pretend, as fome have done, with certainty to fay, what may be the utmoft limits of human fertility.

In an Appendix to the preceding account, with which we have been favoured fince the publication of the volume of the Philofophical

Tranfactions now before us, Dr. Garthfhore has collected fome other inftances of numerous births which we fhall here briefly mention.

The firft of thefe is a cafe of a poor woman in Ayliffe Street, London, who was married in September, 1786, being then in her twenty-eighth year. On the 9th of June (nine months and fix days from the time of her marriage) fhe was delivered, in the evening, of a fmall living girl. No placenta followed, and fhe continued tolerably eafy till the evening of June the 11th, when fhe again fell into labour, and was delivered, about twelve at night, fifty-two hours after the birth of the firft, by the natural pains, of a fecond living girl, larger than the former, and a double placenta foon followed. She continued uneafy all that night; and on Tuefday the 12th, at eleven o'clock in the morning, (eleven hours after the laft labour) fhe was delivered of what the midwife called a falfe conception. On Wednefday the 13th, at three o'clock in the afternoon, fhe was delivered, by natural pains, of a putrid female child, fix inches in length; and on Thurfday the 14th, at feven o'clock in the morning, fhe was delivered of a fourth child, of nearly the fame fize as the third. A double placenta, which feemed to to have belonged to theſe two laſt children, ſoon followed in a putrid ſtate. The birth of theſe four children was ſoon followed by ſymptoms of puerperal fever; and Mr. Gilſon, of White-chapel, being then called to the aſſiſtance of the patient, found her in the moſt dangerous ſituation; but before Mr. Gilſon came both the placenta and the falſe conception were deſtroyed. The midwife deſcribed the latter as a large maſs of ſolid fleſh, covered with a thick ſkin, and meaſuring near ten inches in circumference, but in which, when it was cut through and examined, ſhe could not find the ſmalleſt veſtige of a fœtus. From this deſcription Dr. Garthſhore is at a loſs to determine whether it was only a mere coagulation of blood, or the ſecundines of a fifth conception, in which the fœtus had died ſo early as to be totally diſſolved, and ſo mixed with the coagulated maſs as not to be diſcovered by the midwife.

The patient died on the 17th of June; but the two firſt born children are ſaid to be healthy, and of the uſual ſize of ſmall twins. The eldeſt weighed, in her cloaths, four pounds and one quarter, and the youngeſt ſcarcely four pounds; both together barely the weight of a common full-grown fœtus.

Our

Our author next mentions an account given formerly in the *Journal des Scavans* by M. Seignette, Phyſician at Rochelle, of a woman of Saintonge, who was, at one birth, delivered of nine well-formed children, ſo far advanced that their ſexes could be diſcovered; and he farther obſerves, that Bianchi, in his work *de Naturali in humano corpore vitioſa morboſaque Generatione*, aſſerts, that, in his time, the circumſtance of three children at one birth had happened more than once; that in ſome of the diſtricts of Piedmont there had been ſometimes five at a birth; and that in the dutchy of Milan, a very little time before the year 1741, when his work was publiſhed, ſeven had been born at one time.

In the Harlem Courant for 1755 our author has found an account of a man who was preſented to the Empreſs of Ruſſia, and who was remarkable for being the father of ſeventy-two children by two wives. He likewiſe informs us that the daughter of Gerard Vanderguicht, Engraver to Sir Hans Sloane, confirmed lately to Dr. Combe, a fact he had often heard, that her mother, who is now alive, and in her eighty-ſixth year, had borne thirty-two children

 at at one and thirty births, the laſt being twins, and that they all lived to be chriſtened.

The laſt fact related by Dr. Garthſhore is given on the authority of Mr. Kirwan, a very reſpectable philoſopher, and Fellow of the Royal Society: it relates to a gentleman in Ireland, who was the youngeſt but one of forty ſons, all produced in ſucceſſion from three different wives, by one father, and who all arrived at the age of manhood *.

* The following epitaph, commemorating an inſtance of remarkable fecundity, is inſerted by Mr. Pennant in his Journey to Snowdon:

"Here lyeth the Body of Nicholas Hookes, of Conway, "Gent. who was the 41ſt Child of his Father, William "Hookes, Eſq. by Alice his Wife, and the Father of "twenty-ſeven Children, who died the 20th Day of March, "1637." — EDITOR.

